# The Histology and Histo-Chemistry of Man

**Published:** 1875-06

**Authors:** 


### Books Reviewed.

*The Histology and Histo-Chemistry of Man. A Treatise on the Elements of Composition aud Structure of the Human Body.* By Heinrich Frey, Professor of Medicine in Zurich. Translated by Arthur E. J. Barker, M. D. New York: D. Appleton & Co., 1875. Buffalo: Herger & Ulbrich.

Prof. Frey is favorably known to English readers through the agency of his work on the Microscope and Microscopic Technology, and the admirers of that work will find the present one fully up to their expectations. The study of Histology is too much neglected in the medical curriculum of most of our schools, and but few physicians are prepared to read and appreciate the larger works of Stricker and Rindfleisch. To this class, therefore, the work of Dr. Frey will open a new field for study and research, one in which they will find much pleasure, under the author's direction. The minute anatomy and their chemistry, so far as demonstrated, together with the physiology of tissues, are treated by Dr. Frey in clear and concise terms. The subject matter is handled in an easy, familiar manner, which goes far toward detracting from the dryness which is often complained of in works of this character. The illustrations, which are numerous, are well selected, and seem to elucidate the text in a very acceptable manner. It is a work which ought to receive a large amount of study from American physicians.

